# Ethyl 1-cyclo­propyl-6,7-difluoro-8-meth­oxy-4-oxo-1,4-dihydro­quinoline-3-carboxyl­ate

**DOI:** 10.1107/S1600536808034715

**Published:** 2008-10-31

**Authors:** De-Cai Wang, Xin-Ming Huang, Yan-Ping Liu, Chun-Lei Tang

**Affiliations:** a State Key Laboratory of Materials-Oriented Chemical Engineering, College of Life Sciences and Pharmaceutical Engineering, Nanjing University of Technology, Nanjing 210009, People’s Republic of China

## Abstract

In the title compound, C_16_H_15_F_2_NO_4_, the dihedral angle between the three-membered ring and the quinoline ring system is 64.3 (3)°. In the crystal structure, inter­molecular C—H⋯O hydrogen bonds link the mol­ecules, forming a column running along [101].

## Related literature

The title compound is a key inter­mediate in the synthesis of a series of fluoro­quinolones, see: Matsumoto *et al.* (1996 ▶); Nagano *et al.* (1989 ▶); Petersen *et al.* (1993 ▶).
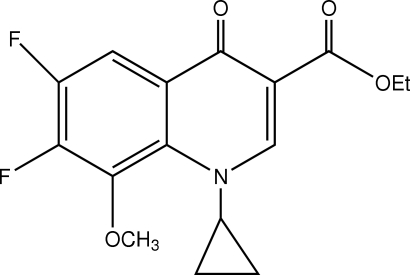


## Experimental

### 

#### Crystal data

C_16_H_15_F_2_NO_4_
*M* *_r_* = 323.29
Monoclinic, 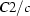

*a* = 16.395 (3) Å
*b* = 17.732 (4) Å
*c* = 12.199 (2) Å
β = 123.71 (3)°
*V* = 2950.1 (14) Å^3^
*Z* = 8
Mo *K*α radiation
μ = 0.12 mm^−1^
*T* = 293 (2) K
0.10 × 0.10 × 0.05 mm

#### Data collection

Enraf–Nonius CAD-4 diffractometer
Absorption correction: ψ scan (North *et al.*, 1968 ▶) *T*_min_ = 0.988, *T*_max_ = 0.994
2760 measured reflections
2663 independent reflections
1580 reflections with *I* > 2σ(*I*)
*R*_int_ = 0.068
3 standard reflections every 200 reflections intensity decay: none

#### Refinement

*R*[*F* ^2^ > 2σ(*F* ^2^)] = 0.061
*wR*(*F* ^2^) = 0.175
*S* = 1.04
2663 reflections
208 parameters
H-atom parameters constrained
Δρ_max_ = 0.21 e Å^−3^
Δρ_min_ = −0.19 e Å^−3^

### 

Data collection: *CAD-4 Software* (Enraf–Nonius, 1989 ▶); cell refinement: *CAD-4 Software*; data reduction: *XCAD4* (Harms & Wocadlo, 1995 ▶); program(s) used to solve structure: *SHELXS97* (Sheldrick, 2008 ▶); program(s) used to refine structure: *SHELXL97* (Sheldrick, 2008 ▶); molecular graphics: *SHELXTL* (Sheldrick, 2008 ▶); software used to prepare material for publication: *PLATON* (Spek, 2003 ▶).

## Supplementary Material

Crystal structure: contains datablocks global, I. DOI: 10.1107/S1600536808034715/is2351sup1.cif

Structure factors: contains datablocks I. DOI: 10.1107/S1600536808034715/is2351Isup2.hkl

Additional supplementary materials:  crystallographic information; 3D view; checkCIF report

## Figures and Tables

**Table 1:** Hydrogen-bond geometry (Å, °)

*D*—H⋯*A*	*D*—H	H⋯*A*	*D*⋯*A*	*D*—H⋯*A*
C1—H1*A*⋯O2^i^	0.96	2.58	3.220 (8)	124
C12—H12*B*⋯O2^ii^	0.97	2.50	3.273 (5)	136

Symmetry codes: (i) 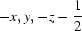; (ii) 
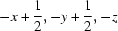
.

**Fig. 1.**
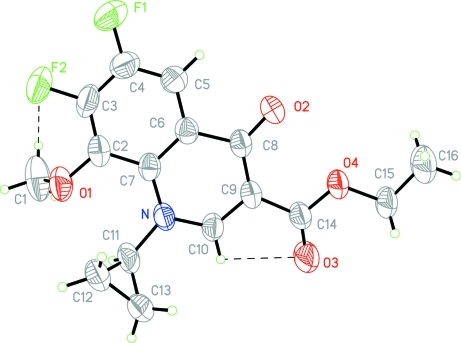
The molecular structure of the title compound, with the atom-numbering scheme. Displacement ellipsoids are drawn at the 30% probability level.

## supplementary crystallographic information

### Comment

The title compound, 1-cyclopropyl-6,7-difluoro-1,4-dihydro-8-methoxy-4-oxo-3-quinolinecarboxylic acid ethyl ester, is a key intermediate to synthesize a series of fluoroquinolones,such as moxifloxacin (Petersen *et al.*, 1993), balofloxacin (Nagano *et al.*, 1989) and gatifloxacin (Matsumoto *et al.*, 1996). As part of our studies in this area, we report here the synthesis and crystal structure of the title compound, (I) (Fig. 1).

The benzene ring and its adjacent six-membered ring were almost coplanar. The dihedral angles between the three-membered ring and the benzene ring is 65.30 (14)°. In the crystal structure, intermolecular C—H···O hydrogen bonds link the molecules, in which they are effective in the stabilization of the structure.

### Experimental

A solution of 26 g (0.075 mol) of 3-cyclopropylamino-2- (2,4,5-trifluoro-3-methoxybenzoyl)acrylic acid ethyl ester and 110 ml of DMF was treated with 22 g (0.16 mol) of K_2_CO_3_, and then heated to 50 °C with stirring for 1 h. The resulting precipitate was filtered, washed with the mixture of ice and water, and dried to give 23 g of the title compound (yield 95%). Crystals of (I) suitable for X-ray diffraction were obtained by slow evaporation of a methanol solution.

### Refinement

All H atoms were placed geometrically (C—H = 0.93–0.98 Å) and refined as riding, with *U*_iso_(H) = 1.2*U*_eq_(C) or 1.5*U*_eq_(methyl C).

### Crystal data

**Table:**

C_16_H_15_F_2_NO_4_	*F*(000) = 1344
*M**_r_* = 323.29	*D*_x_ = 1.456 Mg m^−^^3^
Monoclinic, *C*2/*c*	Mo *K*α radiation, λ = 0.71073 Å
Hall symbol: -C 2yc	Cell parameters from 25 reflections
*a* = 16.395 (3) Å	θ = 9–12°
*b* = 17.732 (4) Å	µ = 0.12 mm^−^^1^
*c* = 12.199 (2) Å	*T* = 293 K
β = 123.71 (3)°	Block, colorless
*V* = 2950.1 (14) Å^3^	0.10 × 0.10 × 0.05 mm
*Z* = 8	

### Data collection

**Table:**

Enraf–Nonius CAD-4 diffractometer	1580 reflections with *I* > 2σ(*I*)
Radiation source: fine-focus sealed tube	*R*_int_ = 0.068
graphite	θ_max_ = 25.2°, θ_min_ = 1.9°
ω/2θ scans	*h* = −2→19
Absorption correction: ψ scan (North *et al.*, 1968)	*k* = 0→21
*T*_min_ = 0.988, *T*_max_ = 0.994	*l* = −14→12
2760 measured reflections	3 standard reflections every 200 reflections
2663 independent reflections	intensity decay: none

### Refinement

**Table:**

Refinement on *F*^2^	Secondary atom site location: difference Fourier map
Least-squares matrix: full	Hydrogen site location: inferred from neighbouring sites
*R*[*F*^2^ > 2σ(*F*^2^)] = 0.061	H-atom parameters constrained
*wR*(*F*^2^) = 0.175	*w* = 1/[σ^2^(*F*_o_^2^) + (0.06*P*)^2^ + 5*P*] where *P* = (*F*_o_^2^ + 2*F*_c_^2^)/3
*S* = 1.04	(Δ/σ)_max_ < 0.001
2663 reflections	Δρ_max_ = 0.21 e Å^−^^3^
208 parameters	Δρ_min_ = −0.19 e Å^−^^3^
0 restraints	Extinction correction: SHELXL97 (Sheldrick, 2008)
Primary atom site location: structure-invariant direct methods	Extinction coefficient: 0.0026 (5)

### Special details

**Table:**

Geometry. All e.s.d.'s (except the e.s.d. in the dihedral angle between two l.s. planes) are estimated using the full covariance matrix. The cell e.s.d.'s are taken into account individually in the estimation of e.s.d.'s in distances, angles and torsion angles; correlations between e.s.d.'s in cell parameters are only used when they are defined by crystal symmetry. An approximate (isotropic) treatment of cell e.s.d.'s is used for estimating e.s.d.'s involving l.s. planes.
Refinement. Refinement of *F*^2^ against ALL reflections. The weighted *R*-factor *wR* and goodness of fit *S* are based on *F*^2^, conventional *R*-factors *R* are based on *F*, with *F* set to zero for negative *F*^2^. The threshold expression of *F*^2^ > σ(*F*^2^) is used only for calculating *R*-factors(gt) *etc*. and is not relevant to the choice of reflections for refinement. *R*-factors based on *F*^2^ are statistically about twice as large as those based on *F*, and *R*- factors based on ALL data will be even larger.

### Fractional atomic coordinates and isotropic or equivalent isotropic displacement parameters (Å^2^)

**Table:**

	*x*	*y*	*z*	*U*_iso_*/*U*_eq_	
N	0.1306 (2)	0.28705 (16)	−0.2762 (2)	0.0512 (7)	
O1	0.1226 (2)	0.17328 (16)	−0.4536 (2)	0.0752 (8)	
F1	0.1209 (2)	−0.01688 (12)	−0.1874 (3)	0.1108 (10)	
C1	0.0365 (4)	0.1548 (3)	−0.5789 (4)	0.1079 (18)	
H1A	−0.0094	0.1957	−0.6088	0.162*	
H1B	0.0072	0.1100	−0.5709	0.162*	
H1C	0.0539	0.1462	−0.6412	0.162*	
O2	0.1328 (2)	0.21459 (14)	0.0430 (2)	0.0736 (8)	
F2	0.1196 (2)	0.02669 (13)	−0.3966 (3)	0.0991 (9)	
C2	0.1223 (2)	0.1543 (2)	−0.3446 (3)	0.0550 (9)	
O3	0.1010 (3)	0.44539 (16)	−0.0591 (3)	0.0988 (11)	
C3	0.1211 (3)	0.0799 (2)	−0.3166 (4)	0.0664 (10)	
O4	0.11605 (19)	0.36229 (14)	0.0869 (2)	0.0666 (7)	
C4	0.1229 (3)	0.0575 (2)	−0.2074 (4)	0.0689 (10)	
C5	0.1266 (3)	0.1091 (2)	−0.1231 (4)	0.0604 (9)	
H5A	0.1274	0.0935	−0.0497	0.073*	
C6	0.1292 (2)	0.18552 (18)	−0.1466 (3)	0.0462 (8)	
C7	0.1284 (2)	0.20942 (18)	−0.2564 (3)	0.0447 (8)	
C8	0.1282 (2)	0.23918 (19)	−0.0548 (3)	0.0466 (8)	
C9	0.1211 (2)	0.31671 (18)	−0.0906 (3)	0.0450 (8)	
C10	0.1234 (2)	0.33541 (19)	−0.1976 (3)	0.0520 (8)	
H10A	0.1196	0.3864	−0.2176	0.062*	
C11	0.1438 (3)	0.3184 (2)	−0.3768 (3)	0.0650 (11)	
H11A	0.0854	0.3183	−0.4672	0.078*	
C12	0.2375 (3)	0.3084 (2)	−0.3630 (4)	0.0737 (11)	
H12A	0.2362	0.3011	−0.4428	0.088*	
H12B	0.2885	0.2807	−0.2869	0.088*	
C13	0.2109 (3)	0.3841 (2)	−0.3402 (4)	0.0821 (13)	
H13A	0.1928	0.4227	−0.4063	0.098*	
H13B	0.2452	0.4024	−0.2502	0.098*	
C14	0.1107 (3)	0.3813 (2)	−0.0227 (3)	0.0551 (9)	
C15	0.1052 (3)	0.4226 (2)	0.1578 (4)	0.0711 (11)	
H15A	0.0395	0.4435	0.1050	0.085*	
H15B	0.1517	0.4626	0.1770	0.085*	
C16	0.1232 (4)	0.3914 (3)	0.2797 (4)	0.0988 (16)	
H16A	0.1172	0.4306	0.3291	0.148*	
H16B	0.1882	0.3706	0.3308	0.148*	
H16C	0.0762	0.3524	0.2595	0.148*	

### Atomic displacement parameters (Å^2^)

**Table:**

	*U*^11^	*U*^22^	*U*^33^	*U*^12^	*U*^13^	*U*^23^
N	0.0654 (18)	0.0554 (18)	0.0445 (15)	0.0109 (13)	0.0378 (14)	0.0036 (13)
O1	0.097 (2)	0.087 (2)	0.0541 (15)	−0.0087 (15)	0.0494 (15)	−0.0196 (13)
F1	0.182 (3)	0.0485 (14)	0.160 (3)	−0.0203 (15)	0.131 (2)	−0.0184 (15)
C1	0.110 (4)	0.158 (5)	0.060 (3)	0.008 (3)	0.049 (3)	−0.025 (3)
O2	0.122 (2)	0.0632 (16)	0.0624 (16)	−0.0063 (15)	0.0677 (17)	−0.0018 (13)
F2	0.144 (2)	0.0714 (16)	0.119 (2)	−0.0169 (15)	0.0963 (19)	−0.0394 (14)
C2	0.051 (2)	0.070 (3)	0.0504 (19)	0.0013 (17)	0.0323 (16)	−0.0102 (17)
O3	0.189 (3)	0.0569 (18)	0.101 (2)	0.0292 (19)	0.112 (2)	0.0115 (16)
C3	0.076 (3)	0.061 (2)	0.075 (2)	−0.0102 (19)	0.050 (2)	−0.029 (2)
O4	0.105 (2)	0.0581 (15)	0.0640 (15)	0.0071 (13)	0.0637 (15)	−0.0046 (12)
C4	0.083 (3)	0.048 (2)	0.097 (3)	−0.0039 (19)	0.063 (2)	−0.003 (2)
C5	0.072 (2)	0.054 (2)	0.073 (2)	−0.0034 (18)	0.052 (2)	−0.0022 (19)
C6	0.0426 (17)	0.053 (2)	0.0471 (17)	−0.0039 (15)	0.0275 (15)	−0.0054 (15)
C7	0.0370 (16)	0.055 (2)	0.0448 (17)	0.0004 (14)	0.0242 (14)	−0.0072 (14)
C8	0.0433 (17)	0.059 (2)	0.0429 (17)	−0.0052 (15)	0.0273 (14)	−0.0085 (15)
C9	0.0409 (17)	0.058 (2)	0.0382 (15)	0.0037 (14)	0.0234 (13)	−0.0034 (15)
C10	0.063 (2)	0.052 (2)	0.0487 (18)	0.0101 (16)	0.0359 (17)	−0.0017 (16)
C11	0.100 (3)	0.066 (2)	0.0420 (18)	0.020 (2)	0.047 (2)	0.0113 (17)
C12	0.106 (3)	0.072 (3)	0.079 (3)	0.010 (2)	0.074 (3)	0.006 (2)
C13	0.143 (4)	0.060 (3)	0.084 (3)	0.005 (3)	0.089 (3)	0.009 (2)
C14	0.072 (2)	0.057 (2)	0.0516 (19)	0.0087 (18)	0.0432 (18)	0.0020 (17)
C15	0.107 (3)	0.064 (2)	0.071 (2)	0.012 (2)	0.068 (2)	−0.0086 (19)
C16	0.145 (4)	0.099 (4)	0.092 (3)	0.014 (3)	0.091 (3)	−0.011 (3)

### Geometric parameters (Å, °)

**Table:**

N—C10	1.340 (4)	C6—C7	1.398 (4)
N—C7	1.402 (4)	C6—C8	1.477 (4)
N—C11	1.468 (4)	C8—C9	1.428 (4)
O1—C2	1.373 (4)	C9—C10	1.367 (4)
O1—C1	1.429 (5)	C9—C14	1.479 (4)
F1—C4	1.345 (4)	C10—H10A	0.9300
C1—H1A	0.9600	C11—C12	1.462 (5)
C1—H1B	0.9600	C11—C13	1.491 (6)
C1—H1C	0.9600	C11—H11A	0.9800
O2—C8	1.233 (4)	C12—C13	1.485 (5)
F2—C3	1.348 (4)	C12—H12A	0.9700
C2—C3	1.365 (5)	C12—H12B	0.9700
C2—C7	1.416 (4)	C13—H13A	0.9700
O3—C14	1.198 (4)	C13—H13B	0.9700
C3—C4	1.374 (5)	C15—C16	1.455 (5)
O4—C14	1.333 (4)	C15—H15A	0.9700
O4—C15	1.448 (4)	C15—H15B	0.9700
C4—C5	1.353 (5)	C16—H16A	0.9600
C5—C6	1.391 (5)	C16—H16B	0.9600
C5—H5A	0.9300	C16—H16C	0.9600
			
C10—N—C7	119.0 (3)	N—C10—H10A	117.0
C10—N—C11	117.9 (3)	C9—C10—H10A	117.0
C7—N—C11	123.1 (3)	C12—C11—N	119.4 (3)
C2—O1—C1	116.6 (3)	C12—C11—C13	60.4 (3)
O1—C1—H1A	109.5	N—C11—C13	118.2 (3)
O1—C1—H1B	109.5	C12—C11—H11A	115.8
H1A—C1—H1B	109.5	N—C11—H11A	115.8
O1—C1—H1C	109.5	C13—C11—H11A	115.8
H1A—C1—H1C	109.5	C11—C12—C13	60.8 (3)
H1B—C1—H1C	109.5	C11—C12—H12A	117.7
C3—C2—O1	119.2 (3)	C13—C12—H12A	117.7
C3—C2—C7	118.8 (3)	C11—C12—H12B	117.7
O1—C2—C7	122.0 (3)	C13—C12—H12B	117.7
F2—C3—C2	119.5 (3)	H12A—C12—H12B	114.8
F2—C3—C4	118.7 (4)	C12—C13—C11	58.8 (3)
C2—C3—C4	121.8 (3)	C12—C13—H13A	117.9
C14—O4—C15	116.7 (3)	C11—C13—H13A	117.9
F1—C4—C5	121.3 (4)	C12—C13—H13B	117.9
F1—C4—C3	118.1 (4)	C11—C13—H13B	117.9
C5—C4—C3	120.6 (4)	H13A—C13—H13B	115.0
C4—C5—C6	119.7 (3)	O3—C14—O4	122.2 (3)
C4—C5—H5A	120.1	O3—C14—C9	124.0 (3)
C6—C5—H5A	120.1	O4—C14—C9	113.7 (3)
C5—C6—C7	120.5 (3)	O4—C15—C16	107.8 (3)
C5—C6—C8	117.2 (3)	O4—C15—H15A	110.1
C7—C6—C8	122.2 (3)	C16—C15—H15A	110.1
C6—C7—N	118.4 (3)	O4—C15—H15B	110.1
C6—C7—C2	118.6 (3)	C16—C15—H15B	110.1
N—C7—C2	123.0 (3)	H15A—C15—H15B	108.5
O2—C8—C9	126.0 (3)	C15—C16—H16A	109.5
O2—C8—C6	119.1 (3)	C15—C16—H16B	109.5
C9—C8—C6	115.0 (3)	H16A—C16—H16B	109.5
C10—C9—C8	119.0 (3)	C15—C16—H16C	109.5
C10—C9—C14	114.9 (3)	H16A—C16—H16C	109.5
C8—C9—C14	126.2 (3)	H16B—C16—H16C	109.5
N—C10—C9	126.0 (3)		
			
C1—O1—C2—C3	66.2 (5)	C5—C6—C8—O2	5.6 (4)
C1—O1—C2—C7	−117.1 (4)	C7—C6—C8—O2	−177.2 (3)
O1—C2—C3—F2	−0.4 (5)	C5—C6—C8—C9	−174.0 (3)
C7—C2—C3—F2	−177.2 (3)	C7—C6—C8—C9	3.1 (4)
O1—C2—C3—C4	178.6 (3)	O2—C8—C9—C10	175.5 (3)
C7—C2—C3—C4	1.8 (6)	C6—C8—C9—C10	−4.9 (4)
F2—C3—C4—F1	−1.4 (6)	O2—C8—C9—C14	−4.8 (5)
C2—C3—C4—F1	179.6 (3)	C6—C8—C9—C14	174.8 (3)
F2—C3—C4—C5	178.6 (3)	C7—N—C10—C9	5.2 (5)
C2—C3—C4—C5	−0.5 (6)	C11—N—C10—C9	−172.8 (3)
F1—C4—C5—C6	179.6 (3)	C8—C9—C10—N	0.9 (5)
C3—C4—C5—C6	−0.4 (6)	C14—C9—C10—N	−178.8 (3)
C4—C5—C6—C7	−0.2 (5)	C10—N—C11—C12	110.6 (4)
C4—C5—C6—C8	177.0 (3)	C7—N—C11—C12	−67.3 (5)
C5—C6—C7—N	179.6 (3)	C10—N—C11—C13	40.6 (5)
C8—C6—C7—N	2.6 (4)	C7—N—C11—C13	−137.3 (3)
C5—C6—C7—C2	1.6 (4)	N—C11—C12—C13	−107.7 (4)
C8—C6—C7—C2	−175.5 (3)	N—C11—C13—C12	109.6 (4)
C10—N—C7—C6	−6.7 (4)	C15—O4—C14—O3	2.7 (5)
C11—N—C7—C6	171.2 (3)	C15—O4—C14—C9	−179.2 (3)
C10—N—C7—C2	171.3 (3)	C10—C9—C14—O3	2.8 (5)
C11—N—C7—C2	−10.8 (5)	C8—C9—C14—O3	−176.9 (4)
C3—C2—C7—C6	−2.3 (5)	C10—C9—C14—O4	−175.2 (3)
O1—C2—C7—C6	−179.0 (3)	C8—C9—C14—O4	5.1 (5)
C3—C2—C7—N	179.7 (3)	C14—O4—C15—C16	−174.3 (3)
O1—C2—C7—N	3.0 (5)		

### Hydrogen-bond geometry (Å, °)

**Table:**

*D*—H···*A*	*D*—H	H···*A*	*D*···*A*	*D*—H···*A*
C1—H1A···O2^i^	0.96	2.58	3.220 (8)	124
C12—H12B···O2^ii^	0.97	2.50	3.273 (5)	136

Symmetry codes: (i) −*x*, *y*, −*z*−1/2; (ii) −*x*+1/2, −*y*+1/2, −*z*.
